# Who First Filled Pulp-Canals?

**Published:** 1902-07

**Authors:** William H. Trueman


### WHO FIRST FILLED PULP-CANALS?

To the Editor:

Sir,—Permit me to suggest that there is no warrant to give Dr. Hudson the credit of being the first to fill pulp-canals, from the fact that the treatment and filling of cases of pulp-exposure, devitalizing the pulp, and treating those cases which are already devitalized and putrescent or abscessed, is fully treated in Bourdet's "Art of the Dentiste," published at Paris, 1757, Chapter III. of the first volume, pages 114 to 132. He quotes from Fauchard, and in Fauchard's first edition, 1728, we have evidence that he also was well informed in regard to the proper methods of meeting these conditions.

Bourdet removed the pulp with an instrument, he tells us, such as watchmakers and engravers use, made of steel, and square. He directs that the temper be partly drawn for a length of about two-thirds of an inch, to prevent its breaking in the canal and to permit its being properly shaped. The end he sharpens, making it three-sided, and with this and the aid of oil of cloves, oil of cinnamon, and spirits of wine, he works down to the apex of the pulp-canal and thoroughly removes its contents. He then places in the canal a little cotton wet with the medicament, and allows it to rest until all irritation has subsided; he then fills with leaves of gold or lead. ("Lorsque la Dent ne fait plus de mal, que le cordon en est détruit, & que le canal est vuidé, il faut garnir exactement la Dent avec des feuilles d'or ou de plomb, & cette Dent se conservera nombre d'années.")

He gives this method, not as something new, but expressly states that it is the usual course of procedure pursued by skilful dentists.

There is no doubt but that Dr. Hudson filled pulp-canals as intimated in the bill, and equally no doubt but that he was so taught by his uncle, as he in turn had been taught by his preceptor. I felt very sad, as a few days ago I stood beside his neglected grave, to know that all Dr. Hudson had learned during his more than thirty years of dental practice was buried in that grave with him. A born leader of men, thoroughly equipped for his life-work, socially and professionally so well qualified to have given the dental profession a masterful uplift, he gave the cold shoulder to those who were struggling to better it, and contributed not one jot or little to make the path easier for those who should follow.

William H. Trueman.

[We are under great obligations to Dr. Wm. H. Trueman for the foregoing, but why, may it be asked, was not this important information given us before? The question has been asked repeatedly, Who first filled pulp-canals? but until now no answer. —Ed.]

